# Dihydroartemisinin (DHA) inhibits myofibroblast differentiation through inducing ferroptosis mediated by ferritinophagy

**DOI:** 10.1016/j.heliyon.2024.e27276

**Published:** 2024-02-28

**Authors:** Ningning Yu, Nan Wang, Weiqun Zhang, Junyu Xue, Quan zhou, Fengai Hu, Xuelian Bai, Naiguo Liu

**Affiliations:** a Medical Research Center, Binzhou Medical University Hospital, Binzhou, 256603, PR China; b Dental Implant Department, Shandong Provincial Hospital Affiliated to Shandong First Medical University, Jinan, 250021, PR China

**Keywords:** Differentiation of myofibroblasts, Ferritinophagy, FTH1, NCOA4, Dihydroartemisinin(DHA), Pulmonary fibrosis

## Abstract

Idiopathic pulmonary fibrosis (IPF) is caused by persistent micro-injuries and aberrant repair processes. Myofibroblast differentiation in lung is a key event for abnormal repair. Dihydroartemisinin(DHA), a well-known anti-malarial drug, have been shown to alleviate pulmonary fibrosis, but its mechanism is not clear. Ferroptosis is involved in the pathgenesis of many diseases, including IPF. Ferritinophagy is a form of cellular autophagy which regulates intracellular iron homeostasis. The function of DHA on myofibroblasts differentiation of pulmonary and whether related with ferroptosis and ferritinophagy are unknown now. Using human fetal lung fibroblast 1(HFL1) cell line and the qRT-PCR, immunofluorescent and Western blotting techniques, we found that after TGF-β1 treatment, the levels of ɑ-SMA expression and ROS increased; the mRNA and protein levels of *FTH1* and *NCOA4,* the content of Fe^2+^ and 4-HNE increased significantly at 6h, then gradually reduced with time. After DHA treatment, FHL1 cells appeared ferroptosis; the levels of α-SMA mRNA and protein reduced and the levels of ROS and 4-HNE increased; the Fe^2+^ levels decreased sharply at 6h, then increased with time, and were higher than normal since 24h; the mRNA and protein levels of *FTH1* and *NCOA4* decreased, exhibited a downward trend. These results show that Fe^2+^, ROS and lipid peroxidation are involved in and ferritinophagy is inhibited during fibroblast-to-myofibroblast differentiation; The depletion of Fe^2+^ at early stage induced by DHA treatment triggers the ferritinophagy in HFL1 cells, leading to degradation of FTH1 and NCOA4 and following increase of Fe^2+^ levels. DHA may inhibit the fibroblast-to-myofibroblast differentiation through inducing ferroptosis mediated by ferritinophagy.

## 1 Introduction

Idiopathic pulmonary fibrosis (IPF) is a progressive interstitial pneumonia characterized by alveolar epithelial changes, focal accumulation of fibroblasts, and excessive deposition of extracellular matrix proteins [1]. Its incidence increases significantly in the recent, and the average life expectancy of patients are 3–5 years after diagnosis. Although some treatments can slow the disease progression, no effective therapeutic measure is found [2]. During the formation of fibrotic foci, fibroblasts accumulate, proliferate, and differentiate into myofibroblasts [3]. Fibroblast-to-myofibroblast differentiation in lung is a key event in the initiation and progression of pulmonary fibrosis [4,5]. The transforming growth factor-β1(TGF-β1) pathway is the main cascade implicated in myofibroblast differentiation [6], and myofibroblast differentiation induced by TGF-β1 has been used as the model of pulmonary fibrosis *in vitro* [7]. α-SMA is a marker protein to assess activated fibroblasts in several tissues and organs including the lung [[8], [9], [10], [11]].

Overload of iron plays a role in the pathogenesis and progression of IPF [12,13]. Iron overloading can participate in generating reactive oxygen species (ROS) directly and indirectly in many diseases, including IPF [[14], [15], [16]]. The process of selective autophagy of ferritin mediated by nuclear receptor coactivator 4(NCOA4), the selective cargo receptor, is referred to as ferritinophagy, which is critical for iron homeostasis [17]. NCOA4 selectively interacts with the key residues of ferritin heavy chain-1 (FTH1) subunit through the conserved C-terminal domain [18]. Ferroptosis mediated by ferritinophagy has been found to be associated with liver fibrosis and neurodegenerative diseases [[19], [20], [21]], and can be used as new strategy for cancer therapy [22,23].

Dihydroartemisinin(DHA) have multiple effects in various diseases. Besides anti-malarial effect, it can promote the death of cells of head and neck carcinoma [22] and lung cancer [24] by inducing ferroptosis. Furthermore, DHA attenuates pulmonary fibrosis induced by bleomycin in rats and myofibroblast-like processes in cultured alveolar epithelial cells (AECs) [25].

The function of DHA on myofibroblast differentiation in pulmonary and whether involved in ferroptosis and ferritinophagy are unclear. In this study, we investigated the role of DHA on myofibroblast differentiation in pulmonary and the mechanisms related with ferroptosis and ferritinophagy, providing the scientific basis for illuminating the pathogensis of pulmonary fibrosis.

## 2 Materials and methods

### 2.1 Cell culture and grouping

Human fetal lung fibroblast (HFL1) (Tongpai Biological Technology Co., LTD, Shanghai, China) were cultured in Ham's F12K medium containing 10% fetal bovine serum (FBS) (Sigma Aldrich, Saint Louis) at 37 °C with 5% CO_2_. When cultured to 70–80% density, cells were randomly divided into four groups, including control group, TGF-β1 treated group, DHA treated group and TGF-β1+ DHA treated group. TGF-β1 treated group was treated with TGF-β1 (6 ng/mL, Sigma Aldrich), and the control group was treated with the same amount of solvent. DHA group were treated with DHA(30 μM, MedChemExpress) [26], and TGF-β1+DHA group were treated with TGF-β1 and DHA at the same time. The morphology of cells were observed under microscope IX51 (Olympus, Tokyo, Japan), and the cells were collected at 6h, 12h, 24h, 36h after treatments respectively and used for further detection.

The benzyloxycarbonyl-Val-Ala-Asp-fluoromethyl ketone (Z-VAD-FMK) [27], necrostatin-1 (Nec-1) [28] and Ferrostatin-1(Fer-1) [29] are specific inhibitor for apoptosis, necroptosis and ferroptosis respetively. To identify the form of death caused by DHA, DHA + Z-VAD-FMK(50 μM, MedChemExpress) [27] treated group, DHA + Nec-1(20 μM, Sigma-Aldrich, USA) [28] treated group, DHA + Fer-1(1 μM, Sigma Aldrich, USA) [29] treated group were replenished.

### 2.2 Cell viability assay

HFL-1 cells were seeded in a 96-well plate at a concentration of 1 × 10^5^ cells/well (100 μL/well) and pre-cultured at 37 °C in 5% CO_2_ for 24 h. At 6h, 12h, 24h and 36h, 10 μL Cell Counting Kit-8 (CCK8) (MedChem Express, China) solution was added in each well and incubated for 1h at 37 °C and 5% CO_2_. The absorbance was measured with a spectral MaxM5 (Eppendorf, Hamburg, Germany) at a wavelength of 450 nm.

### 2.3 Fe^2+^ release assay

HFL1 cells were seeded in a six-well plate, and treated according to the grouping in the "Cell Culture and grouping". The cell culture supernatant was collected at 6h, 12h, 24h and 36h, and the Fe^2+^ concentration was measured with an iron test kit (BioAssay Systems, Hayward, CA94545, USA). Following the instructions of the iron test kit, the sample was prepared and incubated at room temperature for 40 min, and determined the absorbance at the wavelength of 590 nm with the spectrum MaxM5 (Eppendorf, Hamburg, Germany).

### 2.4 ROS assay

After treatments in 6-well plate at a density of 1 × 10^5^ cells/well in 2 mL medium, ROS level was determined by loading 2’,7’-dichlorodihydrofluorescein diacetate (DCFH-DA) (Beyotime Institute of Biotechnology, China) which was diluted to a final concentration of 10 mM with RPMI-1640 culture medium (FBS-free). The fluorescence intensity was monitored with excitation wavelength at 488 nm and emission wavelength at 525 nm used SpectraMax M5 (Eppendorf).

### 2.5 4-HNE analysis

After washing with PBS, the culture containing 2 × 10^6^ cells were resuspended in 250 μL PBS and broken used ultrasonic processor. Supernatants were collected and the levels of lipid peroxidation were determined by reference to a 4-hydroxynonenal (4-HNE) (Elabscience Biotechnology Co., Ltd., Wuhan, China), which were detected following the manufacturers’ instructions. The absorbance of the reaction mixture was measured at 450 nm used SpectraMax M5 (Eppendorf). The results were measured as ng/mL.

### 2.6 mRNA expression analysis

Total RNA was extracted by RNAisoPlus (Takara, Tokyo, Japan), and the purity of the RNA was determined by OD260/OD280. cDNA was synthesized using the PrimescriptRT kit (Perfect RealTime) (Takara, Tokyo, Japan). *α-SMA*, *FTH1*, and *NCOA4* mRNA levels were assessed by quantitative real-time polymerase chain reaction (qRT-PCR) analysis with the SYBR Green Expression Assay (Takara) on a CFX96 Real-Time PCR Detection System (Bio-Rad). PCR conditions were 95 °C for 30 s, followed by 35 cycles of denaturation at 95 °C for 5 s, and annealing at 60 °C for 30 s. The primer sequences used for real-time PCR were as follows: ɑ-SMA gene (NM_001613): forward primer: 5'-AGCGTGGCTATTCCTTCGT-3', reverse primer: 5'-CTCATTTTCAAAGTCCAGAGCTACA--3'; FTH1 gene (NM-002032.3): forward primer: 5'-GCCTCCTACGTTTACCTGTC-3', reverse primer: 5'-GTTTCTCAGCATGTTCCCTC-3'; NCOA4 gene (NM-001145260.2): forward primer: 5'-GATGGCTCATGCTAGTTCAG-3', reverse primer: 5'-AAGGGACAGCTACAATACCG-3'; GAPDH gene (NM-001256799): forward primer: 5'- CAGGAGGCATTGCTGATGAT -3', reverse primer: 5'- GAAGGCTGGGGCTCATTT -3'. Relative expression levels of target gene and reference transcripts were calculated as 2^-△△Ct^. The test of each sample was repeated three times.

### 2.7 Immunofluorescent technique

HFL1 cells were seeded on 14-mm^2^ confocal plate at a density of 5 × 10^4^ cells/mL. The treatments for immunofluorescence were performed at 6 h, 12 h, 24 h and 36 h time points. Cells were washed with PBS, fixed with 100% cold methanol for 5 min, permeabilized with 0.1% Triton X-100 (Absin Bioscience Inc., Shanghai, China) for 10 min, and then blocked with 10% goat serum in PBS buffer for 20 min at room temperature. The plates were incubated overnight at 4°Cwith specific primary antibodies, including anti-FTH1 antibody (Abcam, Cambridge, UK), anti-α-SMA antibody (Abcam, Cambridge, UK) and anti-NCOA4 antibody (Abcam, Cambridge, UK). After washing with PBS, the plates were then routinely stained with a secondary antibody conjugated with goat anti-rabbit IgG-FITC (Boster Biological Technology Co., Ltd.) or goat anti-mouse IgG-TRITC (Boster Biological Technology Co., Ltd.) for 1 h at room temperature. In the end, the nuclei were stained by DAPI (Beyotime Biotechnology, Shanghai, China) for 10 min. Immunofluorescence images were photographed by laser confocal microscopy Leica TCS SP5 (Leica, Mannheim, Germany). Three views were selected randomly for each treatment of every time point, and then the protein expression levels (fluorescence value) were quantified by ImageJ (NIH) software.

### 2.8 Western blot

To determine protein concentration, HFL1 cells were first lysed in a lysis buffer using a BCA kit (Servicebio, Wuhan, China) according to standard protocols. Protein samples were separated with 12% SDS-PAGE and transferred onto PVDF membranes where they were blocked with 5% commercial skim milk at room temperature for 1 h and incubated with primary antibodies for Ferritin heavy chain 1 (FTH1) (1:1000, Abcam, ab65080), and β-actin (1:2000, Boster Biological Technology Co., Ltd. BM3873) overnight at 4 °C. The following morning, the membranes were washed and incubated with HRP secondary antibodies (1:2000, Boster Biological Technology Co., Ltd. BA1054) at room temperature for 1 h. Protein content was detected using ChemiScope 6200Touch multifunctional imaging system(Clinx Science Instruments Co., Ltd. Shanghai, China) and analyzed with software ImageQuantTL. Expression levels of FTH1 protein were expressed as the ratio of FTH1/β-Actin of the same sample for increasing comparability. The FTH1 level in control group at every point was specified as 1 for the convenience of comparison at each time point.

### 2.9 Statistical analysis

Statistical analysis was performed by SPSS 29.0 statistical software (SPSS, Inc.). Statistical analysis was performed using one-way ANOVA and *t*-test. Data are presented as mean ± SEM, and *P* < 0.05 was considered as statistically significant.

## 3 Results

### 3.1 Cell viability of HFL1 cells after different treatments

**Fig. 1:**
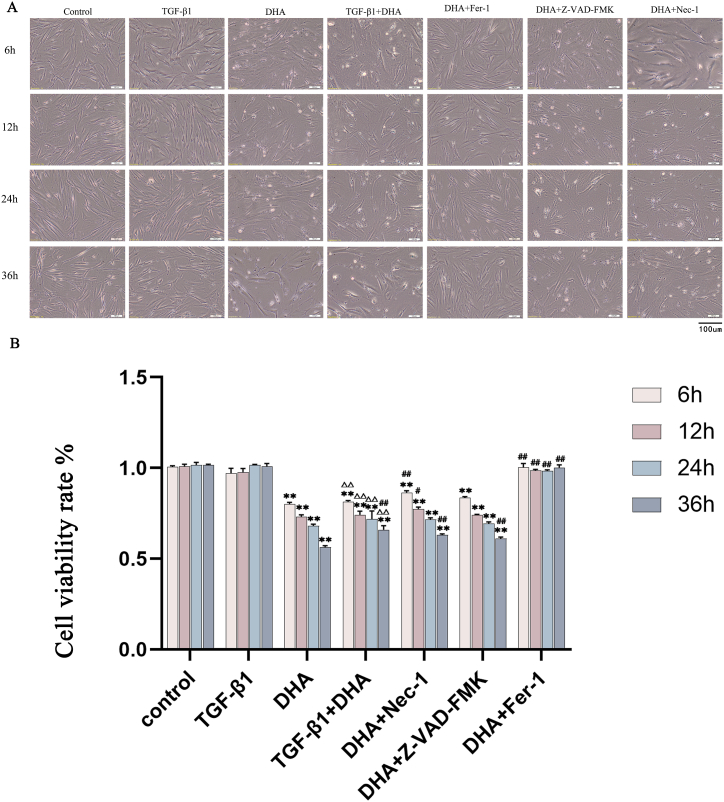
Morphology and cell viability of HFL1 cells in different treatment groups over time. (A) Microscopic images of HFL1cells in different groups over time (200 × , Bar = 100 μm). Cells in DHA, TGF-β1+DHA, DHA + Z-VAD-FMK and DHA + NEC-1 treated groups appeared some dead cells, while no obvious cell death were observed in TGF-β1 and DHA + Fer-1 treated groups. (B) The results were represented as the percentage of the control value. The cell viability decreased significantly after DHA, TGF-β1+DHA, DHA + Z-VAD-FMK and DHA + NEC-1 treatment respectively. Data are presented as mean ± SEM (n = 3). Compared with the control group, **P < 0.01; compared with TGF-β1 treated group, ^△△^*P* < 0.01; compared with DHA treated group, ^#^*P* < 0.05, ^##^*P* < 0.01.

Dead cells were appeared in DHA, DHA + Z-VAD-FMK, DHA + Nec-1 and TGF-β1+DHA treated groups under microscope, while no obvious cell death were found in control group and TGF-β1, DHA + Fer-1 treated groups (Fig. 1A). There were no significant changes of cell viability in the TGF-β1 and TGF-β1+Fer-1 treated groups compared to the control group. However, the cellular viability in the DHA, DHA + Z-VAD-FMK, DHA + Nec-1 and TGF-β1+DHA treated groups decreased significantly compared with the control group (*P* < 0.01). (Fig. 1B). These results showed that only Fer-1 could prevent DHA-induced cell death, while Z-VAD-FMK and Nec-1 did not, indicating that the cell death induced by DHA is ferroptosis, not apoptosis or necroptosis.

### 3.2 Fe^2+^ levels

**Fig. 2:**
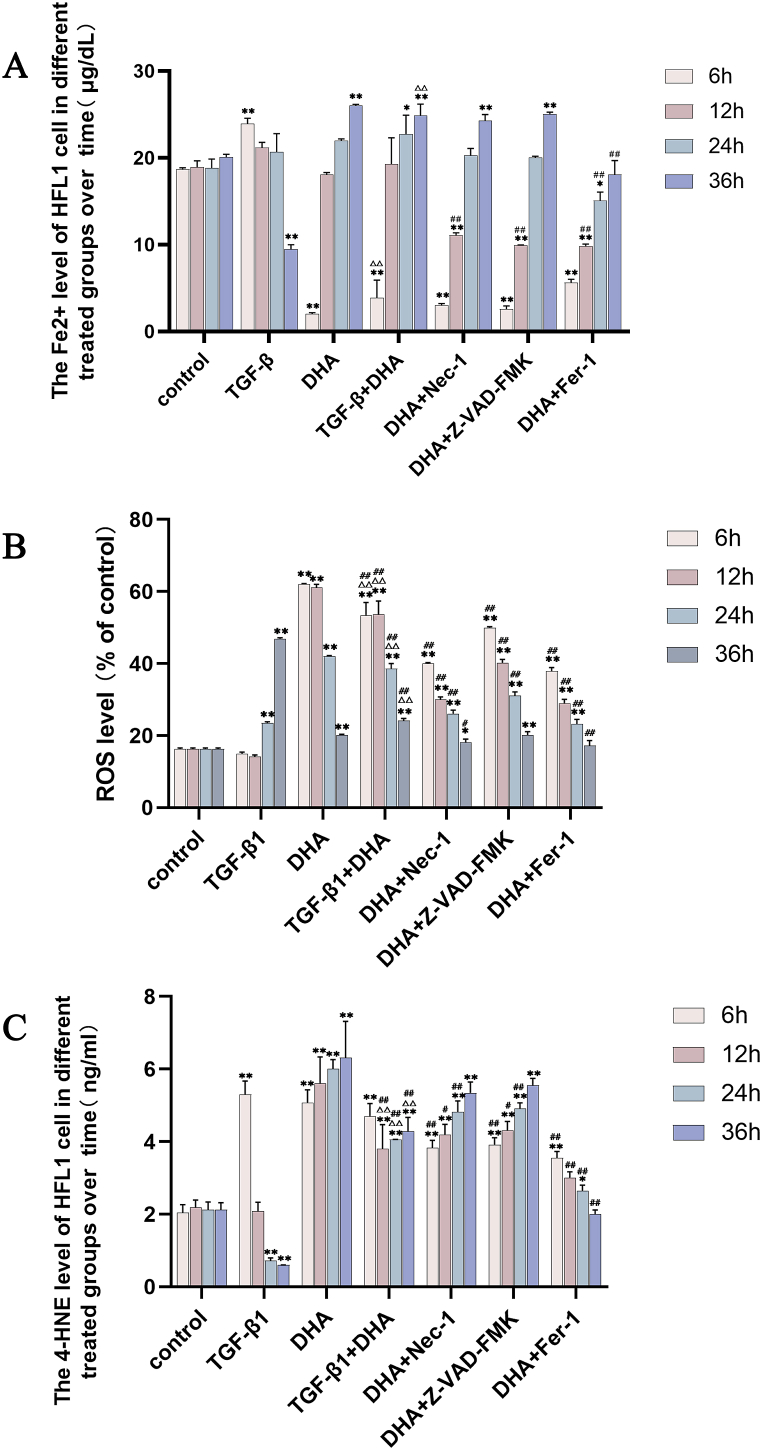
Variation of Fe^2+^, ROS and 4-HNE levels in different treatment groups. (A) Compared with control group, Fe^2+^ levels in TGF-β1-treated group increased at first, then decreased and Fe^2+^ levels after DHA treatment decreased sharply at 6 h and then increased; the variation of Fe^2+^ levels induced by DHA were gradually restored to normal after Fer-1 treatment while not after Z-VAD-FMK and DHA + NEC-1 treatments. (B) Compared with control group, the ROS levels increased with time after TGF-β1 treatment while these in DHA treated group increased sharply at first then decreased, and were higher those in control group till 36h. The variation of ROS levels induced by DHA were alleviated after Z-VAD-FMK, NEC-1 and Fer-1 treatments, and restored to normal only after Fer-1 treatment. (C) Compared with control group, 4-HNE levels increased significantly at 6h, then decreased gradually after TGF-β1 treatment, while these in DHA treated group increased significantly from 6h to 36h. The variation of 4-HNE contents induced by DHA were alleviated after Z-VAD-FMK, and NEC-1, and were restored gradually to normal only after Fer-1 treatment. Data are presented as mean ± SEM (n = 3). Compared with the control group, *P < 0.05, **P < 0.01; compared with TGF-β1 treated group, ^△△^*P* < 0.01; compared with DHA treated group, ^#^*P* < 0.05, ^##^*P* < 0.01.

**Fig. 3:**
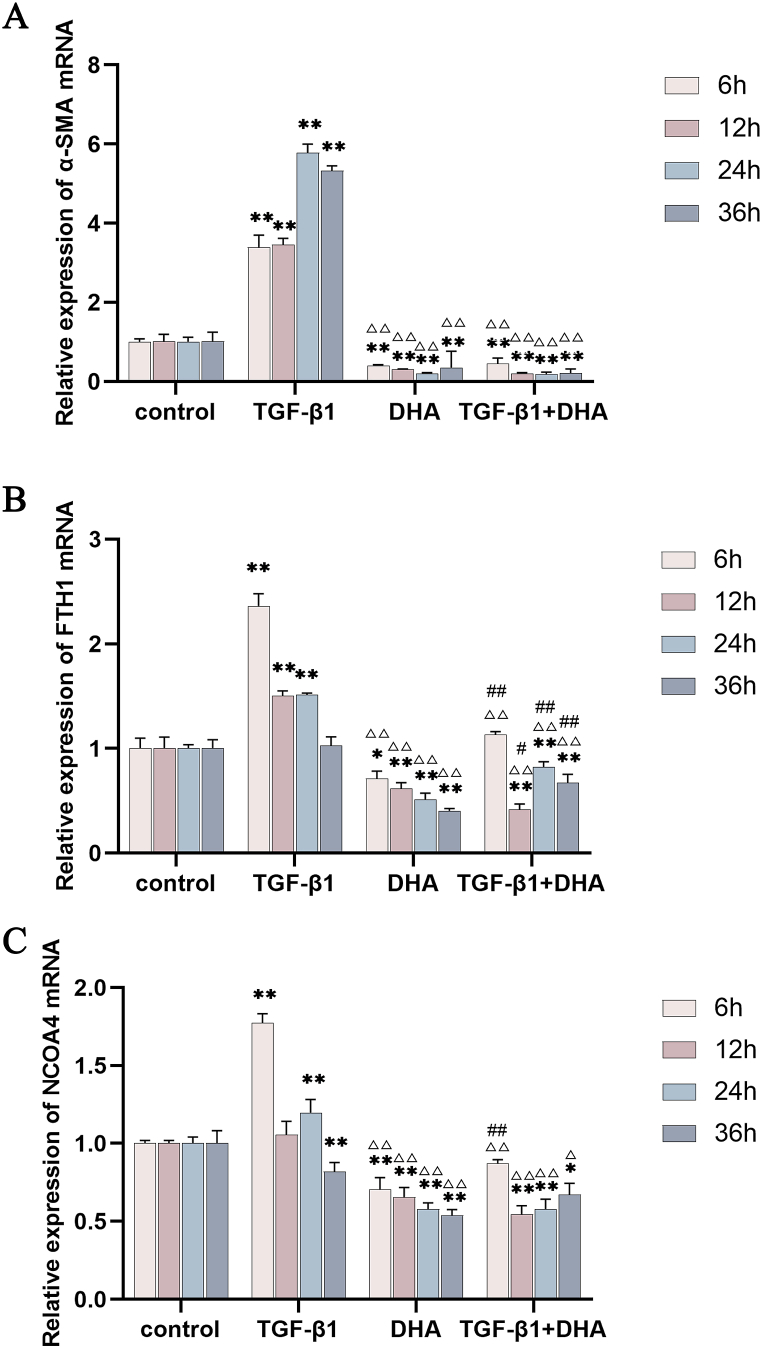
The mRNA expression levels of *α-SMA, FTH I*, and *NCOA4* in different treated groups over time. The levels were assessed by qRT-PCR, and the *GAPDH* was the internal reference. (A) Compared with control group, the expression levels of *α-SMA* mRNAs in TGF-β1 treated group were increased obviously from 6h to 36h, while those were decreased significantly in DHA and DHA + TGF-β1 treated groups. (B) and (C) Compared with control group, the expression levels of *FTH1* and *NCOA4* mRNAs in TGF-β1 treated group increased obviously at early, appearing downward trend in general, while those in DHA and DHA + TGF-β1 treated groups decreased in general. Data are presented as mean ± SEM (n = 3). Compared with the control group, *P < 0.05, **P < 0.01; compared with TGF-β1treated group, ^△^*P* < 0.05, ^△△^*P* < 0.01, compared with DHA treated group, ^#^*P* < 0.05, ^##^*P* < 0.01.

Compared with the control group, Fe^2+^ levels in TGF-β1-treated group increased significantly at 6 h (*P* < 0.01), then gradually decreased with time, and was lower than normal at 36 h (*P* < 0.01). The Fe^2+^ levels in DHA-treated group decreased sharply at 6h, and increased with time from 12h to 36h compared with control group, and were higher than those in control group since 24h. When NEC-1, Z-VAD-FMK and Fer-1 co-treated with DHA respectively, Fe^2+^ levels in all groups showed a sharp decrease at 6h, followed by a rise with time. However, Fe^2+^ levels in DHA + NEC-1 and DHA + Z-VAD-FMK groups were higher than those in control group at 36h, while Fe^2+^ levels in DHA + Fer-1 treated group gradually returned to normal levels. Compared with TGF-β1-treated group, Fe^2+^ level in TGF-β1+DHA group decreased sharply at 6h (*P* < 0.01), and then increased with time, and is higher obviously at 36 h (*P* < 0.01) (Fig. 2A). These results showed that high Fe^2+^ level at late stage is important for ferroptosis (see Fig. 3).

### 3.3 ROS and 4-HNE contents

Compared with the control group, the ROS production in TGF-β1 treated group increased with the time, and the 4-HNE content increased significantly at 6 h, then gradually decreased with time from 12 h to 36h. The ROS and 4-HNE levels in DHA group were higher than those in control group at every time point, but with a downward trend and an upward trend respectively. When NEC-1, Z-VAD-FMK and Fer-1 co-treated with DHA respectively, ROS levels in all groups decreased compared with DHA group at same time point, with a downward trend. ROS level was restored to normal only in DHA + Fer-1 treated group at 36h. 4-HNE levels in DHA + NEC-1 and DHA + Z-VAD-FMK groups were lower than those in DHA group but higher than these in control group at every time point. However, 4-HNE levels in DHA + Fer-1 treated group decreased significantly compared with DHA group and gradually returned to normal levels. Compared with TGF-β1-treated group, the ROS levels in the TGF-β1+DHA treatment group were higher from 6h to 24h, but lower at 36h, showing a downward trend. The 4-HNE contents in TGF-β1+DHA treatment group were significantly increased compared with control and TGF-β1-treated group from 12h to 36h(Fig. 2B, C). These results show that strong lipid peroxidation at later stages is essential for ferroptosis.

### 3.4 The expression levels of α-SMA, FTH1 and NCOA4 mRNAs after different treatments in HFL1 cells

Compared with the control group, the expression level of *α-SMA* mRNA after TGF-β1 treatment significantly increased from 6 h to 36h (*P* < 0.01), and peaked at 24h, while the expression levels of *α-SMA* mRNA decreased obviously at all time points after DHA treatment (FIG. 3A). Compared with the control group, the mRNA expression levels of *FTH1* and *NCOA4* in TGF-β1 treatment group were significantly increased at 6 h (P < 0.01), and last a period, appearing a downward trend, while the expression levels of *FTH1* and *NCOA4* mRNA in DHA group decreased obviously, with a downward trend (FIG. 3B, C). The expression levels of *α-SMA, FTH1* and *NCOA4* mRNA in TGF-β1+DHA treatment group were decreased significantly compared with the control and TGF-β1 treated group (*P* < 0.05) (FIG. 3A, B, C) (see Fig. 6).

### 3.5 The expression levels of α-SMA, FTH1 and NCOA4 proteins after different treatments in HFL1cells

**Fig. 4:**
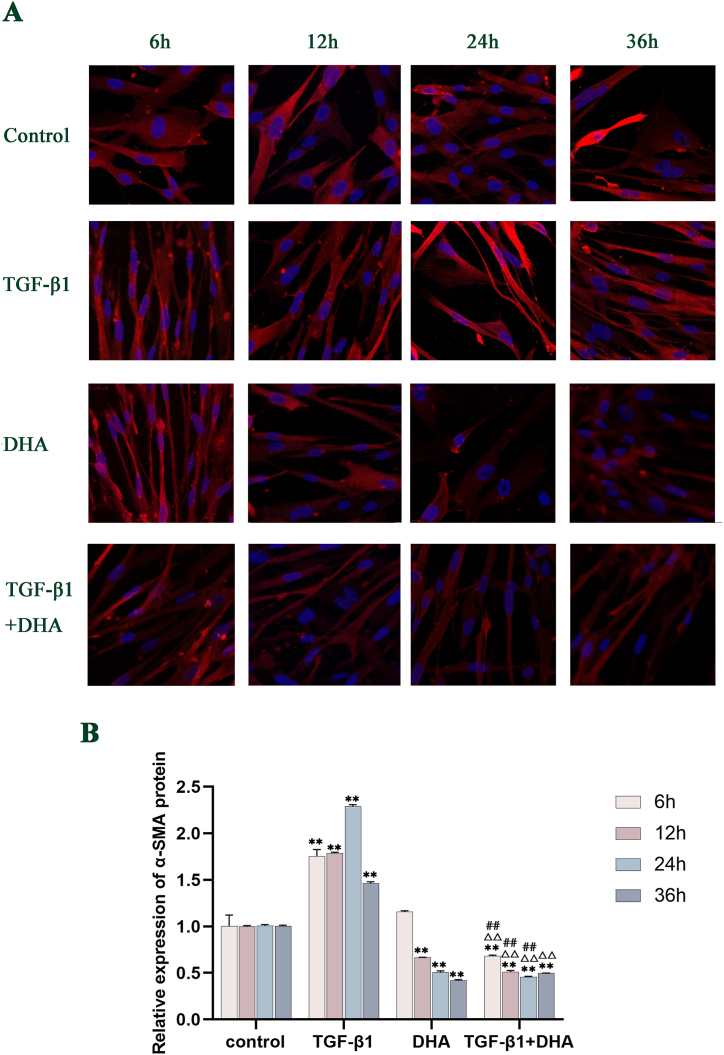
The expression levels of α-SMA protein in different treated groups over time. (A) The expression levels of α-SMA protein in different treated groups of HFL1 cells over time (Immunofluorescence staining). The red signals showed the α-SMA protein expression, and the blue signals showed cell nuclei stained by DAPI. (B) The expression levels of α-SMA protein quantified by image analysis. The expression levels of α-SMA protein were increased in TGF-β1-treated group and decreased after DHA treatment obviously compared with control group. Data are presented as mean ± SEM (n = 3). Compared with the control group, **P < 0.01; compared with TGF-β1treated group, ^△△^*P* < 0.01, compared with DHA treated group, ^##^*P* < 0.01.

**Fig. 5:**
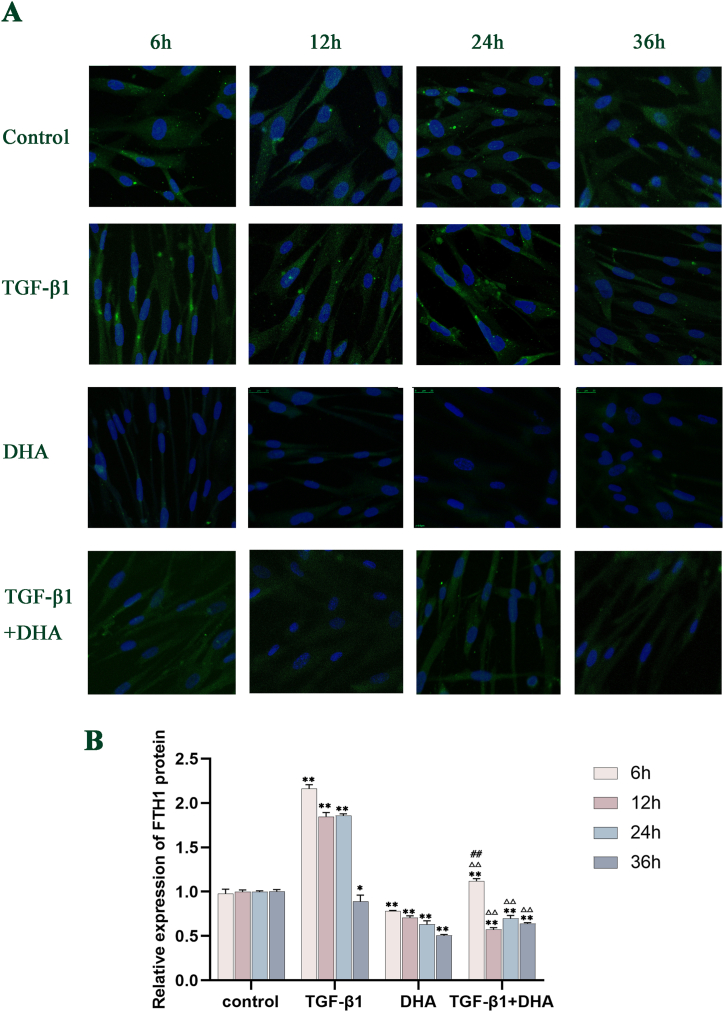
The expression levels of FTH1 protein in different treated groups over time. (A) The expression levels of FTH1 protein in different treated groups of HFL1 cells over time (Immunofluorescence staining). The green signals showed the FTH1 protein expression, and the blue signals showed cell nuclei stained by DAPI. (B) The expression levels of FTH1 protein quantified by image analysis. Compared with control group, the expression levels of FTH1 protein in TGF-β1 group increased obviously from 6h to 24h, while those after DHA treatment decreased significantly at every time point. Data are presented as mean ± SEM (n = 3). Compared with the control group, *P < 0.05, **P < 0.01; compared with TGF-β1treatment group, ^△△^*P* < 0.01, compared with DHA treated group, ^##^*P* < 0.01.

**Fig. 6:**
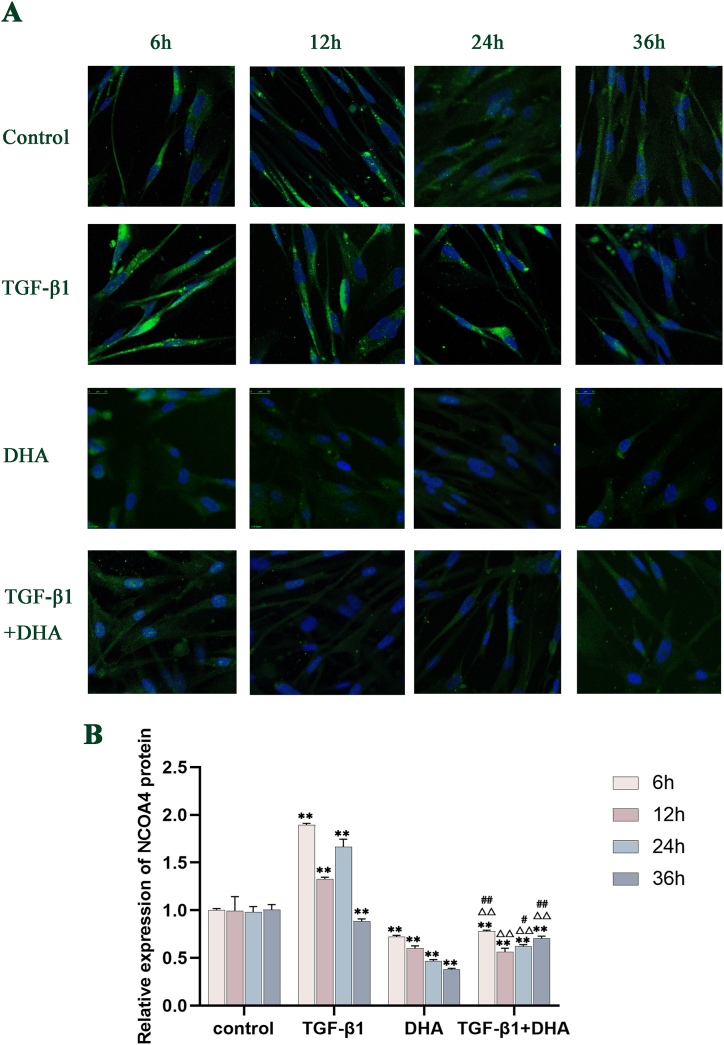
The expression levels of NCOA4 protein in different treated groups of HFL1 cells over time. (A) The expression levels of NCOA4 protein in different treated groups over time (Immunofluorescence staining). The green signals showed the NCOA4 protein expression, and the blue signals showed cell nuclei stained by DAPI. (B) The expression levels of NCOA4 protein quantified by image analysis. Compared with control group, the expression levels of NCOA4 protein in TGF-β1 group increased obviously from 6h to 24h, while those decreased significantly after DHA treatment. The expression levels of protein were significantly decreased after DHA treatment at every time point. Data are presented as mean ± SEM (n = 3). Compared with the control group, **P < 0.01; compared with TGF-β1treatment group, ^△△^*P* < 0.01, compared with DHA treated group, ^#^*P* < 0.05, ^##^*P* < 0.01.

**Fig. 7:**
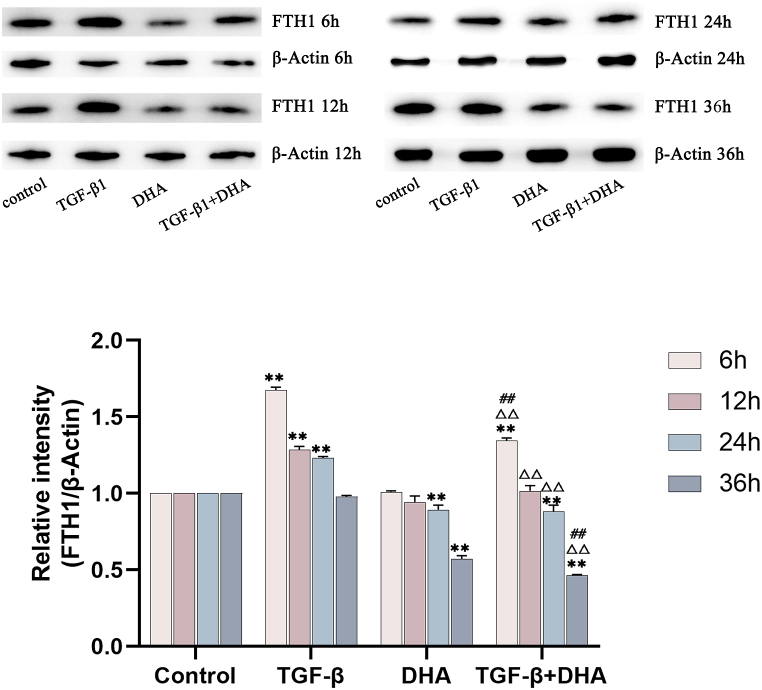
The expression levels of FTH1 protein in different treated groups over time. The expression levels of FTH1 protein in different treated groups of HFL1 cells over time (Western Blot). The β-actin protein was used as an internal reference protein. The relative expression levels of FTH1 protein quantified by image analysis, and corrected by internal reference protein and control. The trend of levels of FTH1 protein expression were consistent with the results of immunofluorescence after TGF-β1 and DHA treatments. Data are presented as mean ± SEM (n = 3). Compared with the control group, *P < 0.05, **P < 0.01; compared with TGF-β1treatment group, ^△△^*P* < 0.01, compared with DHA treated group, ^##^*P* < 0.01.

Compared with the control group, the expression of α-SMA protein in TGF-β1-treated group increased significantly with time from 6 h to 24h (*P* < 0.01), peaked at 24h, and tended to decrease but higher than that in control group at 36 h. The levels of α-SMA protein in DHA group decreased obviously compared with control group from 12h to 36h, with a downward trend. The levels of α-SMA protein in TGF-β1+DHA treated group were significantly lower than those in the control group and TGF-β1-treated group from 6h to 36h(*P* < 0.01) (Fig. 4 A, B). Compared with the control group, the levels of FTH1 and NCOA4 protein in TGF-β1-treated group were significantly higher from 6h to 24h(*P* < 0.01), with a decreasing trend in general. The levels of FTH1 and NCOA4 protein in DHA group decreased with time compared with control group. Compared with the TGF-β1-treated group, the expression levels of FTH1 and NCOA4 proteins in TGF-β1+DHA treatment group were decreased obviously at all points in time (*P* < 0.01). (FIG. 5A, B; FIG. 6A, B, Fig. 7) (see Fig. 5).

## 4 Discussion

Nearly 45% of deaths in developed countries are attributed to some type of chronic fibro-proliferative disease, including IPF and end-stage fibrotic liver, kidney and heart disease [30]. IPF is characterized by fibrosis of the alveoli resulting in restrictive mechanics and impaired gas exchange across the alveolocapillary membrane [31]. Fibroblast-to-myofibroblast differentiation, which is induced by TGF-β1 and characterized by α-SMA expression rise in lung [6,32], is a key event in the initiation and progression of pulmonary fibrosis [4,5]. In our study, the expression levels of α-SMA mRNA and protein were increased compared with those in control group after TGF-β1 treatment, which was consistent with previous report [29], indicating that fibroblast-to-myofibroblast differentiation occurred, and could simulate the development of pulmonary fibrosis to some extent.

Iron overloading was involved in the pathogenesis and progression of pulmonary fibrosis [12,13]. Accumulation of free iron can generate ROS directly and indirectly in the initiation of various diseases, including IPF [[14], [15], [16]]. 4-hydroxy-2-nonena(4-HNE), a marker of lipid peroxidation, which participated in apoptosis, inflammation induction and dysregulation of antioxidant systems, and involved in the initiation and progression of fibrosis [33,34]. Our results showed that the levels of Fe^2+^ and 4-HNE in TGF-β1-treated group increased significantly at 6h compared with the control group, then gradually decreased with time, while ROS content increased with the time after TGF-β1 treatment, and higher than those in control group from 24h, indicating that the excrescent Fe^2+^ and lipid peroxidation play important roles in the early during myofibroblast differentiation, the high ROS level continue involving in myofibroblast differentiation at late stage.

Ferritins are a complex which can accommodate up to 4500 iron atoms and composed of ferritin heavy chain 1 (FTH1) and ferritin light chain (FTL) [35]. FTH1 has ferroxidase activity that promotes iron incorporation and oxidizes Fe^2+^ into the safer Fe^3+^ form [36], which can reduce a large number of free radicals produced by Fe^2+^ [37], and weaken the damage of free radicals to tissues and organs. We found that the expression levels of *FTH1* and *NCOA4* mRNA and proteins in TGF-β1 treated group were increased obviously at 6 h, and kept higher level till 24h, but lower at 36 h compared with the control group. The trends of Fe^2+^ levels, 4-HNE contents, expression levels of *FTH1* and *NCOA4* were consistent after TGF-β1 treatment. These results hinted that levels of Fe^2+^and lipid peroxidation were increased by TGF-β1 treatment in the early, the expression of *FTH1* and *NCOA4* were promoted in respond to high Fe^2+^ level, then Fe^2+^ was oxidized into the safer Fe^3+^ by ferritin, and then the expression of *FTH1* and *NCOA4* gradually reduced following the decrease of Fe^2+^ level. Dihydroquercetin(DHQ) suppressed ferritinophagy by up-regulation of ferritin heavy chain 1 (FTH1), nuclear receptor co-activator 4 (NCOA4) in activated human bronchial epithelial (HBE) cells [38]. In our study, the expression levels of *FTH1* and *NCOA4* were up-regulated in TGF-β1 group at early, and last a period, indicating that ferritinophagy is suppressed during myofibroblast differentiation.

Dihydroartemisinin (DHA), one of the semi-synthetic derivatives of artemisinin (ART), can inhibit inflammation and pulmonary fibrosis induced by bleomycine through suppressing oxidative stress [25,39]. Our study showed that the levels of α-SMA mRNA and protein were decreased after DHA treatment, indicating that DHA can inhibit myofibroblast differentiation and play a role in inhibition of pulmonary fibrosis. Furthermore, our results showed that the levels of ROS and 4-HNE increased, the cell viability decreased after DHA treatment, and appeared dead cells subsequently. The Fe^2+^ levels were higher than normal since 24h after DHA treatment. Excess of Fe^2+^ and ROS, lipid peroxidation are the characteristics of ferroptosis. To confirm the form of cell death, we added the specific inhibitor for apoptosis (Z-VAD-FMK), necroptosis (Nec-1) and ferroptosis(Fer-1) respectively with DHA treatment, and found that only Fer-1 reversed the DHA-induced cell death. These results show that DHA not only inhibit pulmonary fibrosis but also induce ferroptosis of activated HFL1 cells.

Iron stored in ferritin is used during periods of low iron levels and can be liberated from ferritin upon degradation of ferritin in the lysosome [40]. Though ferritin can be degraded by ubiquitin-proteasome [41,42], the most likely and dominant iron release mechanism is lysosomal degradation of ferritin through an autophagic process called ferritinophagy [42,43]. NCOA4 is cargo receptor mediating ferritinophagy, which is important for the selective autophagy of ferritin [17]. NCOA4 could directly interact with ferritin and degrade it in a ferritinophagy-dependent manner, which subsequently released a great amount of iron [44]. Our results showed that Fe^2+^ levels decreased sharply at 6h after DHA treatment, then rose with time, and higher than normal level since 24h; the levels of FTH1 and NCOA4 mRNAs and proteins decreased obviously after DHA treatment. The expression levels of *FTH1* and *NCOA4* mRNA and protein were decreased during the progress of ferritinophagy which was triggered by artesunate in hepinatic stellate cells (HSCs) [20]. These results suggest that DHA decrease Fe^2+^ level sharply at early stage, result in depletion of Fe^2+^ and trigger the ferritinophagy in activated HFL1 cells, leading to degradation of FTH1 and NCOA4 and following increase of Fe^2+^ levels, showing the dual function of DHA on Fe^2+^.

The basal state of iron in cells are important for ferroptosis and that ferroptosis is partially regulated by autophagy and depends on the appropriate lysosomal function, which is important for intracellular iron homeostasis and iron uptake [16]. Dihydroartemisinin (DHA) induced autophagy, which accelerated the degradation of ferritin, increased the labile iron pool, promoted the accumulation of cellular ROS and eventually led to ferroptotic cell death [23]. Artesunate triggered HSC ferroptosis mediated by ferritinophagy was responsible for artesunate-induced anti-fibrosis efficacy [20]. Ferritin degradation via NCOA4-mediated ferritinophagy had been responsible for ferroptosis of bronchial cells in chronic obstructive pulmonary disease (COPD) [45] and in LPS-induced H9c2 myofibroblasts [44]. These researches are consistent with our results and in favor of the conclusion that DHA suppress myofibroblast differentiation through inducing ferroptosis mediated by ferritinophagy.

This study is preliminary and has some limitations. Firstly, there are only *in vitro* experiments but not *in vivo* experiments. It needs to be verified by *in vivo* experiments in animal models in the future. Secondly, gene knockout or knockdown of NCOA4 and FTH1 experiments are also needed for more in-depth studies.

## 5 Conclusion

Our findings provide confirmation that during the progress of differentiation from fibroblasts to myofibroblasts, high Fe^2+^ levels and lipid peroxidation play roles at first, and the ROS production continue to promote myofibroblast differentiation; while ferritinophagy is inhibited. DHA treatment leads to the lack of Fe^2+^ and then triggers the ferritinophagy in activated HFL1 cells, resulting in the degradation of FTH1 and NCOA4 and increasing of Fe^2+^ levels. DHA inhibits the fibroblast-to-myofibroblast differentiation through inducing ferroptosis mediated by ferritinophagy.

## Funding

This work was supported by grants from the 10.13039/501100007129Natural Science Foundation of Shandong Province, No. ZR2019MC019.

## Additional information

No additional information is available for this paper.

## Availability of data and materials

All data are available in the manuscript or the supplementary materials.

## Ethical statement

Only cell line was used as specimen in this study, and it is not involve in the ethical issues.

## CRediT authorship contribution statement

**Ningning Yu:** Writing – original draft, Software, Methodology, Investigation. **Nan Wang:** Methodology, Investigation, Funding acquisition, Data curation. **Weiqun Zhang:** Methodology, Funding acquisition, Formal analysis, Conceptualization. **Junyu Xue:** Visualization, Validation, Formal analysis. **Quan zhou:** Validation, Methodology, Investigation. **Fengai Hu:** Methodology, Investigation. **Xuelian Bai:** Investigation, Formal analysis. **Naiguo Liu:** Writing – review & editing, Supervision, Resources, Project administration, Formal analysis, Conceptualization.

## Declaration of competing interest

The authors declared no potential conflicts of interest with respect to the research, authorship, and/or publication of this article, and no commercial or financial relationships that could be construed as a potential conflict of interest.

## References

[1] Selman M., Pardo A. (2002). Idiopathic pulmonary fibrosis: an epithelial/fibroblastic cross-talk disorder. Respir. Res..

[2] Myall K.J., Kavanagh J.E., Birring S.S. (2019). Idiopathic pulmonary fibrosis-associated cough: mechanisms and management. Pulm. Pharmacol. Ther..

[3] King T.E., Pardo A., Selman M. (2011). Idiopathic pulmonary fibrosis. Lancet.

[4] Wynn T.A. (2008). Cellular and molecular mechanisms of fibrosis. J. Pathol..

[5] Cui H., Banerjee S., Xie N., Ge J., Liu R.M., Matalon S., Thannickal V.J., Liu G. (2016). miR-27a-3p is a negative regulator of lung fibrosis by targeting myofibroblast differentiation. Am. J. Respir. Cell Mol. Biol..

[6] Fernandez I.E., Eickelberg O. (2012). The impact of TGF-beta on lung fibrosis: from targeting to biomarkers. Proc. Am. Thorac. Soc..

[7] Rahaman S.O., Grove L.M., Paruchuri S., Southern B.D., Abraham S., Niese K.A., Scheraga R.G., Ghosh S., Thodeti C.K., Zhang D.X., Moran M.M., Schilling W.P. (2014). TRPV4 mediates myofibroblast differentiation and pulmonary fibrosis in mice. J. Clin. Invest..

[8] Zhang H.Y., Gharaee-Kermani M., Zhang K., Karmiol S., Phan S.H. (1996). Lung fibroblast a-smooth muscle actin expression and contractile phenotype in bleomycin-induced pulmonary fibrosis. Am. J. Pathol..

[9] Carpino G., Morini S., Ginanni Corradini S., Franchitto A., Merli M., Siciliano M., Gentili F., Muda A.O., Berloco P., Rossi M., Attili A.F., Gaudio E. (2005). Alpha-SMA expression in hepatic stellate cells and quantitative analysis of hepatic fibrosis in cirrhosis and in recurrent chronic hepatitis after liver transplantation. Dig. Liver Dis..

[10] Ina K., Kitamura H., Tatsukawa S., Fujikura Y. (2011). Significance of α-SMA in myofibroblasts emerging in renal tubulointerstitial fibrosis. Histol. Histopathol..

[11] Lee H.W., Park Y.M., Lee S.J., Cho H.J., Kim D.H., Lee J.I., Kang M.S., Seo H.J., Shim Y.M., Nam D.H., Kim H.H., Joo K.M. (2013). Alpha-smooth muscle actin (ACTA2) is required for metastatic potential of human lung adenocarcinoma. Clin. Cancer Res..

[12] Puxeddu E., Comandini A., Cavalli F., Pezzuto G., D'Ambrosio C., Senis L., Paci M., Curradi G., Sergiacomi G.L., Saltini C. (2014). Iron laden macrophages in idiopathic pulmonary fibrosis: the telltale of occult alveolar hemorrhage?. Pulm. Pharmacol. Ther..

[13] Ali M.K., Kim R.Y., Brown A.C., Donovan C., Vanka K.S., Mayall J.R., Liu G., Pillar A.L., Jones-Freeman B., Xenaki D., Borghuis T., Karim R., Pinkerton J.W., Aryal R., Heidari M., Martin K.L., Burgess J.K., Oliver B.G., Trinder D., Johnstone D.M., Milward E.A., Hansbro P.M., Horvat J. (2020). Critical role for iron accumulation in the pathogenesis of fibrotic lung disease. J. Pathol..

[14] Kehrer J.P. (2000). The Haber-Weiss reaction and mechanisms of toxicity. Toxicology.

[15] Allden S.J., Ogger P.P., Ghai P., McErlean P., Hewitt R., Toshner R., Walker S.A., Saunders P., Kingston S., Molyneaux P.L., Maher T.M., Lloyd C.M., Byrne A.J. (2019). The transferrin receptor CD71 delineates functionally distinct airway macrophage subsets during idiopathic pulmonary fibrosis. Am. J. Respir. Crit. Care Med..

[16] Nakamura T., Naguro I., Ichijo H. (2019). Iron homeostasis and iron-regulated ROS in cell death, senescence and human diseases. Biochim. Biophys. Acta Gen. Subj..

[17] Mancias J.D., Wang X., Gygi S.P., Harper J.W., Kimmelman A.C. (2014). Quantitative proteomics identifies NCOA4 as the cargo receptor mediating ferritinophagy. Nature.

[18] Dowdle W.E., Nyfeler B., Nagel J., Elling R.A., Liu S., Triantafellow E., Menon S., Wang Z., Honda A., Pardee G., Cantwell J., Luu C., Cornella-Taracido I., Harrington E., Fekkes P., Lei H., Fang Q., Digan M.E., Burdick D., Powers A.F., Helliwell S.B., D'Aquin S., Bastien J., Wang H., Wiederschain D., Kuerth J., Bergman P., Schwalb D., Thomas J., Ugwonali S., Harbinski F., Tallarico J., Wilson C.J., Myer V.E., Porter J.A., Bussiere D.E., Finan P.M., Labow M.A., Mao X., Hamann L.G., Manning B.D., Valdez R.A., Nicholson T., Schirle M., Knapp M.S., Keaney E.P., Murphy L.O. (2014). Selective VPS34 inhibitor blocks autophagy and uncovers a role for NCOA4 in ferritin degradation and iron homeostasis in vivo. Nat. Cell Biol..

[19] Zhang Z., Yao Z., Wang L., Ding H., Shao J., Chen A., Zhang F., Zheng S. (2018). Activation of ferritinophagy is required for the RNA-binding protein ELAVL1/HuR to regulate ferroptosis in hepatic stellate cells. Autophagy.

[20] Kong Z., Liu R., Cheng Y. (2019). Artesunate alleviates liver fibrosis by regulating ferroptosis signaling pathway. Biomed. Pharmacother..

[21] Zhang Z., Wang X., Wang Z., Zhang Z., Cao Y., Wei Z., Shao J., Chen A., Zhang F., Zheng S. (2021). Dihydroartemisinin alleviates hepatic fibrosis through inducing ferroptosis in hepatic stellate cells. Biofactors.

[22] Lin R., Zhang Z., Chen L., Zhou Y., Zou P., Feng C., Wang L., Liang G. (2016). Dihydroartemisinin (DHA) induces ferroptosis and causes cell cycle arrest in head and neck carcinoma cells. Cancer Lett..

[23] Du J., Wang T., Li Y., Zhou Y., Wang X., Yu X., Ren X., An Y., Wu Y., Sun W., Fan W., Zhu Q., Wang Y., Tong X. (2019). DHA inhibits proliferation and induces ferroptosis of leukemia cells through autophagy dependent degradation of ferritin. Free Radic. Biol. Med..

[24] Yuan B., Liao F., Shi Z.Z., Ren Y., Deng X.L., Yang T.T., Li D.Y., Li R.F., Pu D.D., Wang Y.J., Tan Y., Yang Z., Zhang Y.H. (2020). Dihydroartemisinin inhibits the proliferation, colony formation and induces ferroptosis of lung cancer cells by inhibiting PRIM2/SLC7A11 Axis. OncoTargets Ther..

[25] Yang D.X., Qiu J., Zhou H.H., Yu Y., Zhou D.L., Xu Y., Zhu M.Z., Ge X.P., Li J.M., Lv C.J., Zhang H.Q., Yuan W.D. (2018). Dihydroartemisinin alleviates oxidative stress in bleomycin-induced pulmonary fibrosis. Life Sci..

[26] Yao Y., Guo Q., Cao Y., Qiu Y., Tan R., Yu Z., Zhou Y., Lu N. (2018). Artemisinin derivatives inactivate cancerassociated fibroblasts through suppressing TGF-β signaling in breast cancer. J. Exp. Clin. Cancer Res..

[27] Fransolet M., Noël L., Henry L., Labied S., Blacher S., Nisolle M., Munaut C. (2019). Evaluation of Z-VAD-FMK as an anti-apoptotic drug to prevent granulosa cell apoptosis and follicular death after human ovarian tissue transplantation. J. Assist. Reprod. Genet..

[28] Liu M., Li H., Yang R., Ji D., Xia X. (2022). GSK872 and necrostatin-1 protect retinal ganglion cells against necroptosis through inhibition of RIP1/RIP3/MLKL pathway in glutamate-induced retinal excitotoxic model of glaucoma. J. Neuroinflammation.

[29] Gong Y., Wang N., Liu N., Dong H. (2019). Lipid peroxidation and GPX4 inhibition are common causes for myofibroblast differentiation and ferroptosis. DNA Cell Biol..

[30] Wynn T.A. (2007). Common and unique mechanisms regulate fibrosis in various fibroproliferative diseases. J. Clin. Invest..

[31] Sgalla G., Iovene B., Calvello M., Ori M., Varone F., Richeldi L. (2018). Idiopathic pulmonary fibrosis: pathogenesis and management. Respir. Res..

[32] Kang H. (2017). Role of MicroRNAs in TGF-beta signaling pathway-mediated pulmonary fibrosis. Int. J. Mol. Sci..

[33] Zhang H., Forman H.J. (2017). Signaling by 4-hydroxy-2-nonenal: exposure protocols, target selectivity and degradation. Arch. Biochem. Biophys..

[34] Reyes-Jiménez E., Ramírez-Hernández A.A., Santos-Álvarez J.C., Velázquez-Enríquez J.M., Pina-Canseco S., Baltiérrez-Hoyos R., Vásquez-Garzón V.R. (2021). Involvement of 4-hydroxy-2-nonenal in the pathogenesis of pulmonary fibrosis. Mol. Cell. Biochem..

[35] Finazzi D., Arosio P. (2014). Biology of ferritin in mammals: an update on iron storage, oxidative damage and neurodegeneration. Arch. Toxicol..

[36] Zarjou A., Jeney V., Arosio P., Poli M., Zavaczki E., Balla G., Balla J. (2010). Ferritin ferroxidase activity: a potent inhibitor of osteogenesis. J. Bone Miner. Res..

[37] Timoshnikov V.A., Kobzeva T.V., Polyakov N.E., Kontoghiorghes G.J. (2015). Inhibition of Fe^2+^- and Fe^3+^- induced hydroxyl radical production by the iron-chelating drug deferiprone. Free Radic. Biol. Med..

[38] Yuan L., Sun Y., Zhou N., Wu W., Zheng W., Wang Y. (2022). Dihydroquercetin attenuates silica-induced pulmonary fibrosis by inhibiting ferroptosis signaling pathway. Front. Pharmacol..

[39] Yang D., Yuan W., Lv C., Li N., Liu T., Wang L., Sun Y., Qiu X., Fu Q. (2015). Dihydroartemisinin supresses inflammation and fibrosis in bleomycine-induced pulmonary fibrosis in rats. Int. J. Clin. Exp. Pathol..

[40] Asano T., Komatsu M., Yamaguchi-Iwai Y., Ishikawa F., Mizushima N., Iwai K. (2011). Distinct mechanisms of ferritin delivery to lysosomes in iron-depleted and iron-replete cells. Mol. Cell Biol..

[41] Zhang Y., Mikhael M., Xu D., Li Y., Soe-Lin S., Ning B., Li W., Nie G., Zhao Y., Ponka P. (2010). Lysosomal proteolysis is the primary degradation pathway for cytosolic ferritin and cytosolic ferritin degradation is necessary for iron exit. Antioxidants Redox Signal..

[42] Biasiotto G., Di Lorenzo D., Archetti S., Zanella I. (2016). Iron and neurodegeneration: is ferritinophagy the link?. Mol. Neurobiol..

[43] Ndayisaba A., Kaindlstorfer C., Wenning G.K. (2019). Iron in neurodegeneration-cause or consequence?. Front. Neurosci..

[44] Li N., Wang W., Zhou H., Wu Q., Duan M., Liu C., Wu H., Deng W., Shen D., Tang Q. (2020). Ferritinophagy-mediated ferroptosis is involved in sepsis-induced cardiac injury. Free Radic. Biol. Med..

[45] Yoshida M., Minagawa S., Araya J., Sakamoto T., Hara H., Tsubouchi K., Hosaka Y., Ichikawa A., Saito N., Kadota T., Sato N., Kurita Y., Kobayashi K., Ito S., Utsumi H., Wakui H., Numata T., Kaneko Y., Mori S., Asano H., Yamashita M., Odaka M., Morikawa T., Nakayama K., Iwamoto T., Imai H., Kuwano K. (2019). Involvement of cigarette smoke-induced epithelial cell ferroptosis in COPD pathogenesis. Nat. Commun..

